# Dislocation of the Femur into the Sacro-Ischiatic Notch, Reduced by Manipulation

**Published:** 1857-11

**Authors:** G. R. Henry

**Affiliations:** Burlington, Iowa


ART. VII.—*A Case of Dislocation of the Femur into the Sacro-Ischiatic Notch. Reduced by Manipulation.* By G. R. HENRY, M.D., of Burlington, Iowa.

On the 24th inst., I was requested by Dr. W. W. Nassau to visit with him a man who had been thrown from a rail car on our Western road.

I found a very robust, muscular man, about 40 years old, with his right leg very much everted and shortened two inches and a half, presenting the indications of a fracture of the neck. From the absence of crepitus, and the obstinate persistence of shortening, we concluded that there was no fracture; and from our inability to feel the head of the bone and the position of the trochanter, we diagnosed a dislocation into the sacro-ischiatic notch.

We determined to attempt the reduction without applying the pulleys. After administering chloroform, we flexed the knee upon the thigh, and gradually elevated the latter upon the body, then bent it downwards and towards the other side, keeping up manipulations upon the trochanter. During its descent, the head of the bone was felt, and heard to move from the notch and take its position upon the dorsum ilii. After repeating this proceeding two or three times, it slipped into the acetabulum. We were much aided and assisted by Drs. Peet and McCann.

The ease with which the reduction was accomplished, the slight laceration of the muscles, the comfortable condition of the patient afterwards, in connection with the rapid recovery from all ill effects of the injury, satisfy me that, *where it is practicable*, this mode of procedure is far preferable to the one in common practice.

